# Influence of Mabs on PrP^Sc^ Formation Using *In Vitro* and Cell-Free Systems

**DOI:** 10.1371/journal.pone.0041626

**Published:** 2012-07-27

**Authors:** Binggong Chang, Robert Petersen, Thomas Wisniewski, Richard Rubenstein

**Affiliations:** 1 Departments of Neurology and Physiology/Pharmacology, State University New York Downstate Medical Center, Brooklyn, New York, United States of America; 2 Departments of Pathology, Neuroscience, and Neurology, Case Western Reserve University, Cleveland, Ohio, United States of America; 3 Departments of Neurology, Psychiatry and Pathology, New York University School of Medicine, New York, New York, United States of America; National Institute for Agricultural Research, France

## Abstract

PrP^Sc^ is believed to serve as a template for the conversion of PrP^C^ to the abnormal isoform. This process requires contact between the two proteins and implies that there may be critical contact sites that are important for conversion. We hypothesized that antibodies binding to either PrP^c^or PrP^Sc^ would hinder or prevent the formation of the PrP^C^–PrP^Sc^ complex and thus slow down or prevent the conversion process. Two systems were used to analyze the effect of different antibodies on PrP^Sc^ formation: (i) neuroblastoma cells persistently infected with the 22L mouse-adapted scrapie stain, and (ii) protein misfolding cyclic amplification (PMCA), which uses PrP^Sc^ as a template or seed, and a series of incubations and sonications, to convert PrP^C^ to PrP^Sc^. The two systems yielded similar results, in most cases, and demonstrate that PrP-specific monoclonal antibodies (Mabs) vary in their ability to inhibit the PrP^C^–PrP^Sc^ conversion process. Based on the numerous and varied Mabs analyzed, the inhibitory effect does not appear to be epitope specific, related to PrP^C^ conformation, or to cell membrane localization, but is influenced by the targeted PrP region (amino vs carboxy).

## Introduction

Prion diseases are a group of fatal neurodegenerative disorders that are associated with conformational conversion of the cellular prion protein, PrP^C^, which is mainly α-helical with very few beta sheets, into a β-sheet-rich form, PrP^Sc^ [1]–[5]. The mechanism by which PrP^C^ is converted to the abnormal isoform is still not clear, but it is presumed to involve a PrP^C^–PrP^Sc^ complex, with the latter serving as a conformational template [6]. In this model, PrP^Sc^ serves as a template that binds to PrP^C^ and produces a conformational conversion into the abnormal isoform. This raises the issue of whether there are critical contact sites that mediate conversion. If this is the case, interfering with or blocking complex formation should prevent the PrP^C^ to PrP^Sc^ conversion process. Previous reports have described anti-PrP antibodies that can stop or hinder the conversion process add reference 44 and renumber [7]–[14].

Protein misfolding cyclic amplification (PMCA) is an assay that mimics the PrP^Sc^ propagation process under cell-free conditions. In this method PrP^Sc^ is amplified by converting PrP^C^ to a PrP^Sc^ seed during incubation with periodic sonication [15]. PrP^Sc^ generated by PMCA is infectious in wild-type animals [16] and can be indefinitely propagated while preserving the properties of the original PrP^Sc^ strain [16]–[18]. Furthermore, PMCA has been quite useful in studying the cofactors that influence PrP conversion [19]–[26], and in detecting PrP^Sc^ from biological samples of humans and animals [17], [27]–[34].

We hypothesized that antibodies binding to PrP^c^ and/or PrP^Sc^ might hinder or prevent the formation of the PrP^C^–PrP^Sc^ complex and thus prevent the conversion process. We compared the effect of individual PrP-specific monoclonal antibodies (Mabs) on the PrP^C^–PrP^Sc^ conversion process using both an N2a/22L cell culture model and the test-tube PMCA system. Our results demonstrate that the Mabs have a range of inhibitory effects on the PrP^C^–PrP^Sc^ conversion process. The degree of inhibition is Mab specific and more dependent on the antibody targeting region than on the specific epitope being recognized. Furthermore, since the PMCA-based method is dose-dependent and rapid, it may serve as an ideal screening assay for potential inhibitors of both PrP^Sc^ accumulation and the progression of prion diseases.

## Methods

### Animals (Ethics Statement)

All procedures involving animals and their care were conducted in accordance with the United States Department of Agriculture Animal Welfare Act and the National Institute of Health policy on Humane Care and Use of Laboratory Animals. Tissue samples from uninfected and prion agent-infected mice and hamsters were obtained using protocols approved by the Institutional Animal Care and Use Committee of the SUNY Downstate Medical Center (protocol #’s 07-250-09 and 07-251-09).

### PMCA and Western Blotting

A 10% normal hamster brain homogenate (NBH) was prepared in phosphate buffered saline (PBS) containing 1% Triton X-100, 4mM EDTA and 1% protease inhibitor cocktail (Abcam). PrP-specific Mabs were generated against recombinant (murine or hamster) PrP or brain-derived proteinase K (PK)-resistant purified PrP^Sc^ [35] from brains of clinical mice infected with the ME7 mouse-adapted scrapie strain or clinical hamsters infected with the 263K hamster-adapted scrapie strain. The Mabs used in this study were purified (Montage Antibody Purification kit; Millipore, Billerica, CA), isotyped (ELISA Mouse Antibody Isotyping kit; Thermo Fisher, Rockford, IL), and epitope mapped (Table 1). The immunoreactivity of all the Mabs were analyzed on western blots against denatured, PK-digested and undigested PrP derived from uninfected and infected brain homogenates as well as by ELISA against recombinant PrP. With the exception of Mab 3F4, each of the individual Mabs had equivalent immunoreactivity against murine and hamster PrP^Sc^ on an immunoglobulin concentration basis. All of the Mabs were highly reactive against both hamster PrP^C^ and PrP^Sc^ isoforms and, for the PMCA studies, were individually added to the 10% NBH at a final concentrations of 50 µg/ml. A 10% 263K brain homogenate was prepared in PBS only and diluted to a final concentration of 10^−4^. A 100 µl aliquot of this homogenate was initially combined with 10 µl of 10% NBH (with or without added Mab). Each sample was sonicated (QSONIC at 480W power, 60 Amplitude, 40,000 J energy, 90 sec process time, 3 sec pulse on−1 sec pulse off), then incubated at 37°C for 1 hr. This was defined as one cycle of serial PMCA (sPMCA). At the completion of each cycle, an additional 10 µl of 10% NBH (with or without Mab) was added. At the end of every five cycles, 100 µl of the total volume was transferred to a new tube containing an equal volume of 10% NBH (with or without Mab) and the cycling reactions continued. At the completion of 40 cycles (sPMCA_40_), 500 µl from each sample was PK-treated (100 µg/ml final concentration, 50°C, 30 min), followed by the addition of protease inhibitor cocktail. The sample was heated (100°C, 10 min) and then centrifuged at 16,000×g for 2 min at room temperature. The supernatant was combined with 6X Laemmli sample buffer, and 50 µl was electrophoresed in a 12% sodium dodecyl sulfate polyacrylamide gel electrophoresis (SDS-PAGE) followed by transfer to nitrocellulose membrane. The membrane was blocked for 1 hr in PBS containing 0.1% Tween 20 (PBST) with 5% non-fat dry milk and incubated with 2 µg/ml biotinylated Mab 08-6/2F11. The membrane was washed 3 times (10 min each) with PBST, incubated for 60 min in HRP-conjugated streptavidin (Invitrogen) (1∶5000 in PBST containing 5% non-fat dry milk) followed by 3 additional PBST washes and detection of proteins with ECL Supersignal West Dura kit (Thermo Fisher). Quantification of PrP^Sc^ was performed by densitometric analysis using NIH Image J software.

**Table 1:** Characterization of Mabs.

Mab		Isotype	Epitope	Immunoreactivity	
				Mse PrP^C^/PrP^Sc^	Ham. PrP^C^/PrP^Sc^
08-6/7E4	(7E4)	IgG1	aa 29–35	+/+	+/+
01-7/10E4	(10E4)	IgG1	aa 54–89	+/+	+/+
08-1/5D6	(5D6)	IgG2b	aa 91–101	+/+	+/+
08-1/11F12	(11F12)	IgG2b	aa 91–111	+/+	+/+
3F4		IgG2a	aa 107–112	−/−	+/+
03-9/8E11	(8E11)	IgG2a	aa 112–120	+/+	+/+
02-3/3A2	(3A2)	IgG1	aa 121–125	+/+	+/+
08-1/8E9	(8E9)	IgG2b	aa 135–153	+/+	+/+
01-16/1B11	(1B11)	IgG2a	aa 141–145	+/+	+/+
01-7/2F7	(2F7)	IgG1	aa 193–210	+/+	+/+

### Epitope Mapping

Cellulose membranes spotted with 99 overlapping 13-mer PrP peptides were produced as previously described [36]. The membranes were blocked with 5% non-fat dry milk/tris-buffered saline containing 0.1% Tween 20 (TBST) probed with antibody diluted 1∶5000 in 1% normal goat serum/TBS at 4°C overnight, followed by horseradish peroxidase (HRP)-conjugated goat anti-mouse secondary (Cappel 55570) for 2 hours at room temperature, and detected using Millipore Immobilon Western chemiluminescent HRP substrate (Cat WBKLS0500). Membranes were regenerated for re-use by shaking with dimethylformamide for 30 minutes, then 8M urea/50mM Tris-HCl pH 8.0/1% β-mercaptoethanol (β-MC)/1%SDS overnight at 37°C, followed by a 30 min wash in the same buffer, and then twice for 30 minutes each in 50% methanol/glacial acetic acid, and finally three times for 5 minutes each in methanol. After air drying membranes were stored in a sealed container at room temperature.

### Infection and Mab Treatment of N2a Cells

Murine neuroblastoma N2a cells (ATCC line CCL 131) were grown in the Minimal Essential Medium supplemented with 10% FBS, penicillin and streptomycin and infected with 2% 22L brain homogenate as described previously [12]. Following infection, the amount of PrP^Sc^ in 200 µg cell lysate aliquots of the N2a/22L cells was determined by PK digestion (1 µg/µl PK for 30 min at 37°C), SDS-PAGE on 12.5% Tris-tricine gels [37] and western blot analysis as previously described [12].

For treatment of cells with Mabs, N2a/22L cells (from the fifth passage after infection and higher) were plated in six-well plates and once the cells were 70–80% confluent, Mabs were added at a final concentration of 10 µg/ml and incubation was continued for 96 hr. Each Mab was tested in three independent experiments using independently infected cell lines. Each experiment included both a positive control (untreated N2a/22L cells) and a negative control (N2a cells), which were subjected to PK digestion. The level of PK-resistant PrP^Sc^ was measured in western blots using HRP-conjugated sheep anti-mouse IgG as the secondary reagent and ECL Supersignal West Dura kit. Membranes were exposed to X-ray film (X-Omat Blue XB-1; Kodak, New Haven, CT,) with a constant exposure time of 30 sec. The films were converted into eight-bit grayscale digital files. Quantification of PrP^Sc^ was performed by densitometric analysis using NIH Image J software v. 1.34. Areas under the curves for three PrP bands representing non-, mono-and diglycosylated isoforms of the protein were summarized from each sample to calculate the total amount of PrP and expressed as percentages of the average value from a positive control (untreated N2a/22L), whereas the optic density of the background was taken from negative control lanes (N2a cells).

## Results

**Figure 1:**
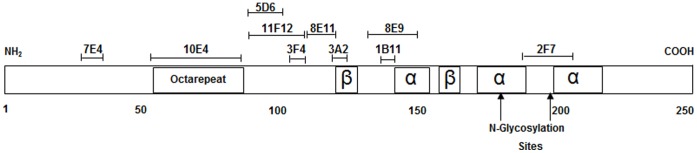
Linear diagram of prion protein showing epitope location of PrP-specific monoclonal antibodies used on N2a/22L cells and in PMCA.

The PrP-specific Mabs that were evaluated for their ability to prevent PrP^C^ to PrP^Sc^ conversion have linear epitopes that span the entire prion protein from the amino to the carboxy terminus (Table 1, Fig. 1).

**Figure 2:**
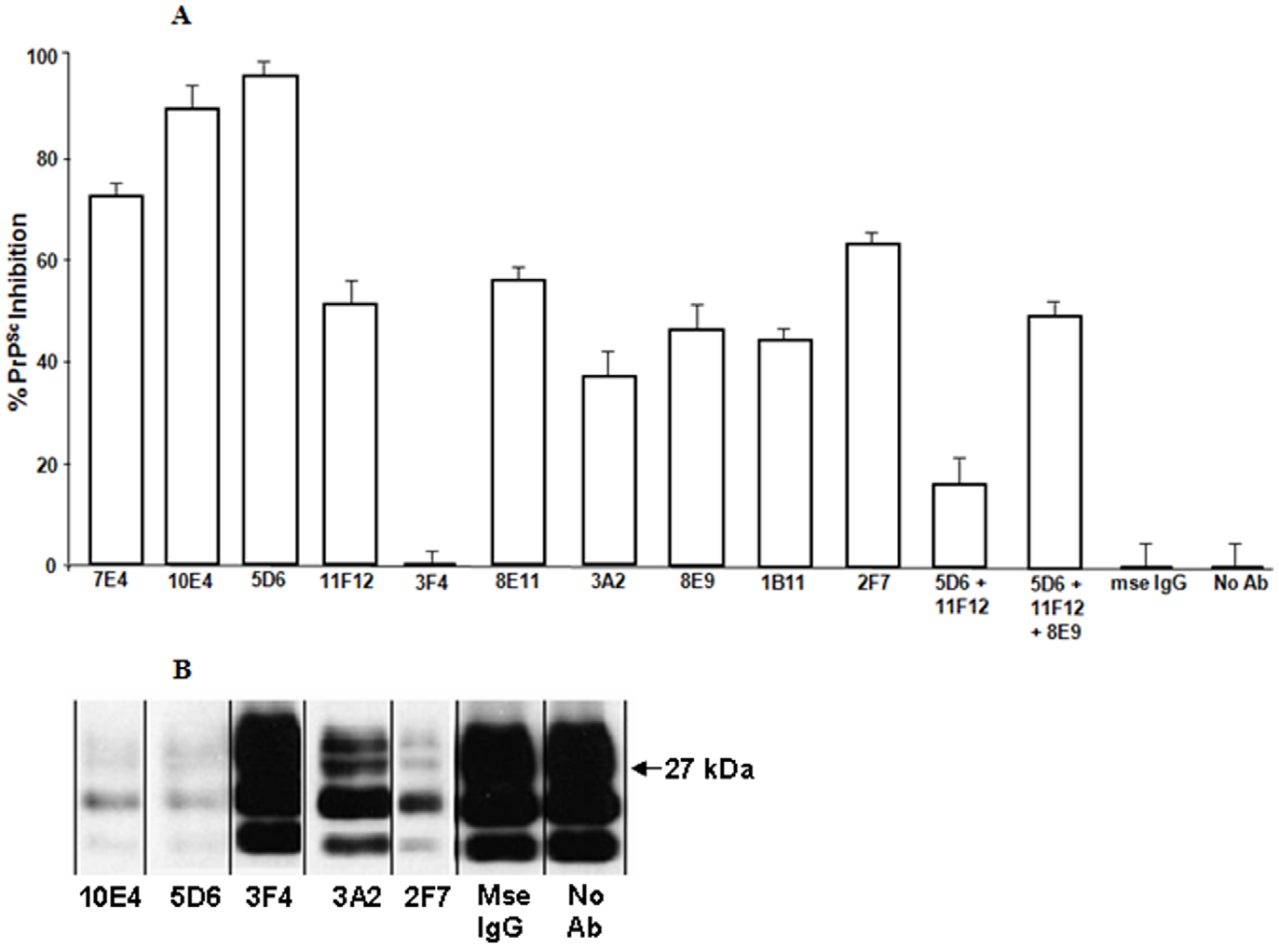
A. Mab Inhibition of PrP^Sc^ in N2a/22L Cells. N2a/22L cells were incubated with purified Mabs for 96 hrs. Cells were harvested and PK-treated lysates were western blotted (**see** **Figure 2B** **for representative western blots**). PrP^Sc^ western blots were quantitated and the amount of inhibition was determined relative to N2a/22L control cultures. The controls consisted of cells both in the absence of Mab and in the presence of normal mouse IgG. The % PrP^Sc^ inhibition plotted represents the mean ± SD from three independent experiments as described in Methods.

We used N2a cells persistently infected with the 22L mouse-adapted scrapie strain (N2a/22L) to evaluate the affect of each Mab on PrP^Sc^ formation (Fig. 2A and 2B). Treatment with the Mabs did not result in any cytotoxicity to the N2a/22L cells throughout the incubation period. Further, incubation of the. N2a/22L cells with 10 µg/ml purified, irrelevant mouse IgG had no effect on PrP^Sc^ formation compared to untreated N2a/22L cultures (Figs. 2A and 2B). Mab 3F4 did not reduce PrP^Sc^ formation compared to control N2a/22L cultures lacking Mab. Mab 3F4 does not react with mouse prion protein so this was not surprising [38]. The ability of a singly added Mab to inhibit PrP^Sc^ formation was not related to a specific epitope since all of the remaining singly added Mabs inhibited PrP^Sc^ formation to varying degrees. Of the individually added Mabs, 5D6 was the most effective at inhibiting PrP^Sc^ formation (95% inhibition) while 3A2 was the least effective (38% inhibition). Targeting the amino terminus with Mab 7E4 was effective at inhibiting 73% PrP^Sc^ formation as was targeting the octapeptide repeat region using Mab 10E4 which resulted in almost 90% inhibition. Strangely, although their epitopes overlap, Mab 11F12 was less effective than Mab 5D6 at inhibiting PrP^Sc^ formation (53% vs 95% inhibition). This is in contrast to Mabs 8E9 and 1B11, which have overlapping epitopes with 8E9 being more expansive, and resulted in 52% and 42% inhibition, respectively. The combination of 5D6 and 11F12 did not result in an additive inhibitory effect and, in fact, resulted in less inhibition than either one alone. This was confirmed in studies where the addition of 8E9 to 5D6 and 11F12 caused a 45% PrP^Sc^ inhibition, which was slightly better than 8E9 alone, although the predicted additive inhibitory effect of 63% for the three Mab combination (48% for 8E9 plus 15% for the 5D6 and 11F12 combination) was not observed.

**Figure 3:**
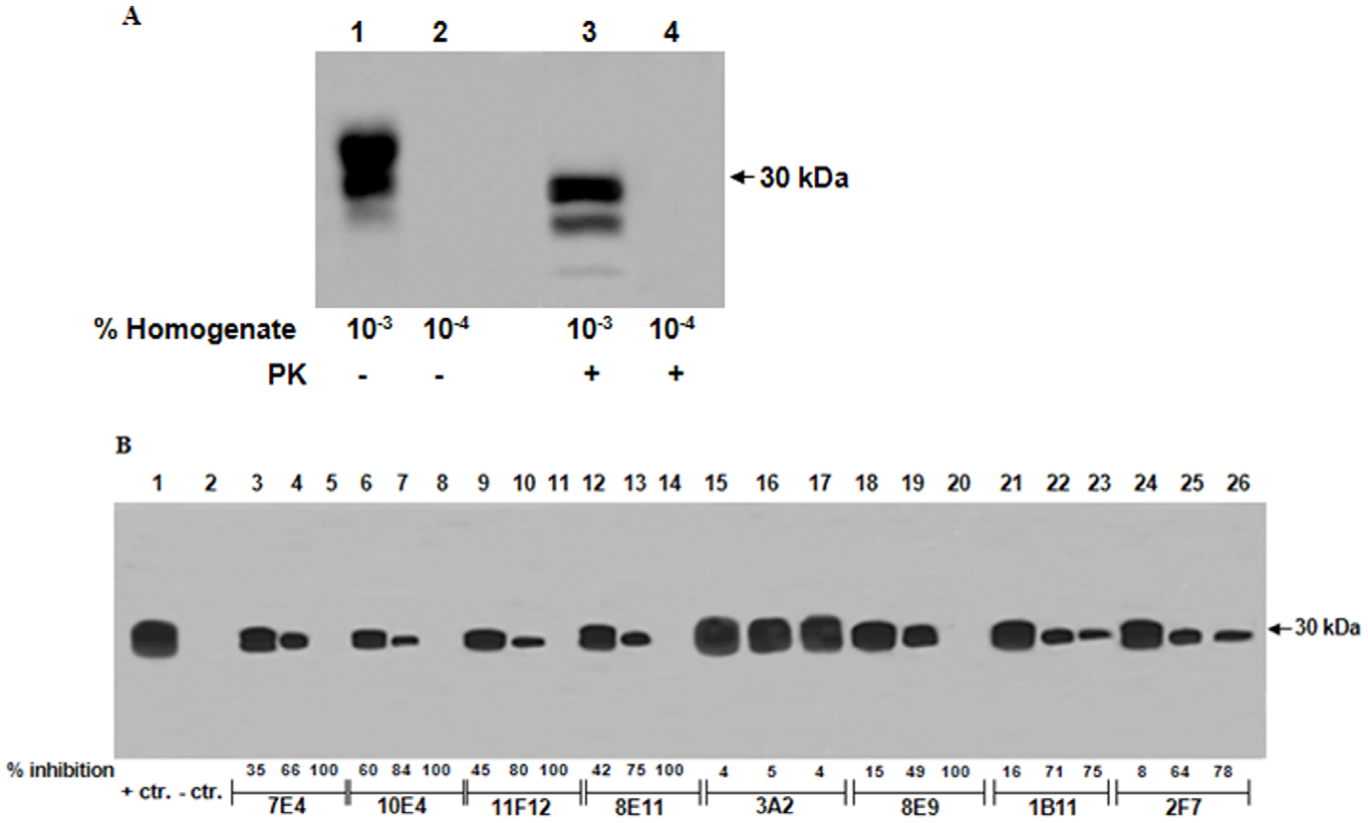
A. Western blot of 263K brain homogenate that was used as the seed for PMCA. Dilutions of the brain homogenate was prepared and either untreated (lanes 1 and 2) or PK-treated (lanes 3 and 4) prior to SDS-PAGE and western blotting. A 10−3 dilution prior to and after PK demonstrates the three protein banding pattern typical for 263K brain homogenate whereas no bands are visible at a 10−4 dilution of the same homogenate. **B.** Western blotting of the PMCA products following sPMCA40 in the absence and presence of PrP-Specific Mabs. Fourty cycles of serial PMCA was carried out in the absence or presence of Mabs as described in the text. Each Mab was added at a final concentration of 12, 25, and 50 µg/ml. Following PK treatment, the PMCA products were subjected to SDS-PAGE, western blotted and immunostained for PrPSc. The protein bands were quantitated and the level of PrPSc inhibition, relative to the no Mab and normal mouse IgG controls, were determined.

Studies were performed with PMCA to determine whether a cell-free system can recapitulate the effect of Mabs on PrP^C^ conversion observed in infected cells. This system also allowed us to evaluate whether accessibility of Mab to membrane associated PrP^C^ in the living cells influences the PrP^C^ to PrP^Sc^ conversion process. Mabs (12–50 µg/ml final concentration) were added throughout the sPMCA_40_ protocol along with the 10% NBH spiked with a 10^−4^ dilution of 263K infected brain homogenate as described in the Methods section. This dilution of infected brain homogenate does not result in detectable PK resistant PrP^Sc^ immunostaining (Fig. 3A) and, therefore, did not interfere with the detection of newly formed PrP^Sc^. At the completion of sPMCA_40_, the samples were digested with PK (100 µg/ml) and analyzed on immunoblots using biotinylated Mab 2F11 which reacts equally with both hamster PrP^C^ and PrP^Sc^. It is interesting to note that although the 263K-infected brain homogenate displayed the 3 band pattern typical for the multiple glycosylated forms of PrP^C^ and PrP^Sc^ (Fig. 3A), the sPMCA_40_ products in the positive controls and Mab-treated reactions consisted of only a single diglycosylated 30 kDa PrP^Sc^ band observed after PK digestion at the higher levels of inhibition, >50%, but had two bands or a smear when there was less inhibition (Fig. 3B).

PMCA in the presence of Mabs was also used to study the importance of binding site specificity in the PrP^C^ to PrP^Sc^ conversion process (Fig. 3). We performed sPMCA with different Mab concentrations to determine the minimum amount of Mab necessary to inhibit the conversion process. Using a 10^−4^ dilution of 263K-infected hamster brain homogenate as the PrP^Sc^ seed and a 10% normal brain homogenate (NBH) as the source of PrP^C^, we tested the ability of Mabs to inhibit the conversion of PrP^C^ to PrP^Sc^. For each Mab, final concentrations of 12 µg/ml (lanes 3, 6, 9, 12, 15, 18, 21 and 24), 25 µg/ml (lanes 4, 7, 10, 13, 16, 19, 22 and 25) and 50 µg/ml (lanes 5, 8, 11, 14, 17, 20, 23, and 26) were prepared in hamster NBH and used in the sPMCA reactions. Compared to sPMCA_40_, which contained no Mab (lane 1) and with the exception of 02–3/3A2, the majority of the PrP-specific Mabs inhibited the conversion process in a dose-related manner although some were more effective than others. Mabs 7E4, 10E4, 11F12, 8E11, and 8E9 completely inhibited the conversion process at 50 µg/ml while Mabs 1B11 and 2F7 inhibited the conversion process to a lesser degree. The inhibition caused by the Mabs was a specific response since sPMCA_40_ studies replacing Mabs with purified normal mouse IgG (at 12–50 µg/ml) in the 10% NBH did not cause any inhibition of PrP^Sc^ formation (data not shown). It is interesting to note that, with the exception of only 8E9, the epitopes for all the Mabs that caused complete inhibition are located in the amino half of the PrP while those that caused incomplete inhibition are located in the carboxy half of PrP. There was good correlation between the extent of PrP^Sc^ inhibition when 10 µg/ml Mab in cell culture was compared to 12 µg/ml Mab with sPMCA_40_.

**Figure 4:**
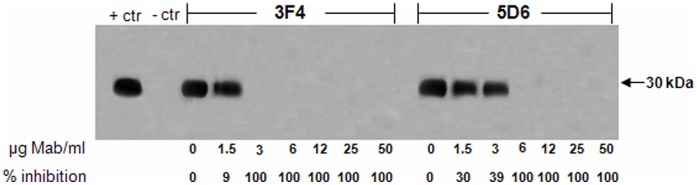
Influence of Mabs 3F4 and 5D6 on PrP^Sc^ formation following sPMCA_40_. Mabs 3F4 and 5D6 were added to sPMCA_40_ at final concentrations of 0–50 µg/ml. The PMCA products were PK treated and western blotted. The PrP^Sc^ was quantitated and the level of PrP^Sc^ inhibition was determined relative to control reactions.

A separate study using sPMCA_40_ demonstrated that Mabs 3F4 and 5D6 caused complete inhibition of PrP^Sc^ formation at 12–50 µg/ml (Fig. 4). Therefore we extended those studies and evaluated the effects of Mabs 3F4 and 5D6 using a wider range of Mab concentrations (1.5–50 µg/ml). Compared to the other antibodies in this study, Mabs 3F4 and 5D6 had the most pronounced effects on PrP^Sc^ formation as demonstrated by the low concentrations of 3 and 6 µg/ml, respectively, causing complete inhibition (Fig. 4). The potent inhibitory effect of 5D6 on PrP^Sc^ observed using sPMCA_40_ coincides with its dramatic effect in the N2a/22L culture model. Furthermore, the poor PrP^Sc^ inhibition by 3A2 with sPMCA_40_ (Fig. 3B) corresponded well with the poor inhibition (only 32% reduction compared to negative control) observed in the cell culture system (Fig. 2A and 2B).

## Discussion

Currently, there is no effective treatment for prion diseases. To date, hundreds of chemical compounds have been identified that antagonize prion propagation *in vitro* in cell culture-based assays and/or *in vivo* in animal studies [39]–[43]. Unfortunately, many compounds efficient in *in vitro* studies were only effective in animal models if treatment was begun before or close to the time of inoculation with the infectious agent [44]. Furthermore, many of the candidate compounds have limited usefulness clinically due to toxicity or their inability to cross the blood-brain barrier [e.g. Congo red [45], iododoxorubicin, β-sheet breakers].

Additional therapeutic and/or prophylactic strategies have been and continue to be pursued. Vaccination with recombinant mouse PrP delays the onset of prion disease in mice [46]. Passive immunization with anti-PrP antibodies was shown not only to inhibit formation of PrP^Sc^ in a cell-free system [47], but was also shown to prevent infection of susceptible N2a cells [7] and to inhibit prion replication in infected cells [8], [47], [48]. The effectiveness of these treatments were also dependent on when they were administered relative to the time of infection.

In an initial passive immunization study using wild-type CD1 mice, Mabs 8B4 (to mouse PrP residues 34–52) and 8H4 (to mouse PrP residues 175–185) given immediately after challenge with 139A scrapie by intraperitoneal (IP) injection (50 µg/week), resulted in a significant prolongation of the incubation period with 10% of the 8B4 treated animals remaining disease free in the group challenged with a lower dose of PrP^Sc^ [10]. In another study using higher antibody doses (4000 µg/week IP) of either ICSM 18 (to mouse PrP residues 146–158) or ICSM 35 (to mouse residues 95 to 105), prion infection from a peripheral source was completely prevented if treatment was continued for 7 or 30 days immediately following PrP^Sc^ challenge [9]. Furthermore, a transgenic mouse model that expresses Mab 6H4 is resistant to prion infection via IP injection by a mechanism that involves either perturbation of cellular PrP trafficking/PrP^C^ degradation or disruption of the PrP^C^–PrP^Sc^ interaction [49].

Previous studies have reported that the 132–140 portion of PrP^C^ [8] or the 132–156 region of PrP [50]–[53] are important for the generation of PrP^Sc^. Rigter et al. [54] found two high affinity binding regions for protein-protein interactions using ovine peptide-arrays: (i) sheep-PrP peptides 43–102, including the amino-terminal octarepeats, and (ii) sheep-PrP peptides 134–177 which encompasses most of the scrapie susceptibility-associated polymorphisms in sheep. Moroncini et al. [55] found that residues within the 89–112 and 136–158 segments of PrP^C^ are key components of the PrP^C^–PrP^Sc^ complex. Beringue et al. [11] reported that antibodies exclusively binding PrP^C^ were relatively inefficient inhibitors of PrP^Sc^ accumulation compared with antibodies that additionally recognize disease-associated PrP isoforms. Féraudet et al. [56] screened 145 anti-PrP Mabs for their capacity to inhibit PrP^Sc^ replication in infected N2a or Rov9 cells. They identified four different linear epitopes that hindered the PrP^C^ to PrP^Sc^ conversion: the amino terminal region 26–35, the octarepeat region 59–89, the intermediate region 97–102, and the central region 130–160. The observation that antibodies that bind to the amino terminus of the prion protein are capable of inhibiting conversion suggests that the endogenous proteolytic cleavage occurs after the site of conversion.

To more completely explore the possible therapeutic effect of anti-PrP antibodies, and to establish another system to analyze the influence of Abs on the conversion process, we screened Mabs produced in our laboratory for their capacity to inhibit PrP^Sc^ formation. This screening was performed using N2a/22L cells and cell-free sPMCA. In N2a/22L cultures, all Mabs that react with mouse PrP reduce PrP^Sc^ formation although with varying efficiency. Thus, similar to previous results [55], we found that the ability to inhibit PrP^C^ to PrP^Sc^ conversion was not restricted to a single epitope or limited to a specific region of the protein. However, the greatest inhibition was observed with Mabs that targeted epitopes in the amino terminal, unstructured region of the PrP. The greatest inhibition in the N2a/22L cells was with Mab 5D6. This is consistent with a prior study using Mab 6D11 (anti-PrP residues 95–105) which in a screen of multiple Mabs, only one produced the greatest inhibition (∼100%) [12]. Mab 6D11 has also been shown to have some efficacy *in vivo* prolonging the pre-symptomatic incubation period [57]. The Mab inhibition results obtained using PMCA were similar to that found in the cell culture system. PMCA has the advantages over the cell culture model of being cost-effective, simple, rapid, sensitive, and more amenable to studies of dose dependence. For identification of potential candidate Mabs that might have *in vivo* activity it is likely that such Mabs would have to produce 90 to 100% inhibition in the much simpler *in vitro* systems.

The interaction of PrP^C^ to PrP^Sc^ is critically dependent on the structural compatibility of the molecules as supported by the existence of a species barrier for prion infection, related to minor differences in the primary sequence of PrP^C^ in different species. Therefore, it is not surprising that antibodies that may alter or mask the critical epitopes on PrP^C^ and/or PrP^Sc^, involved during the mutual conformational complementarity required in prion propagation, will be inhibitory for prion replication. Although many anti-PrP antibodies targeting different regions of PrP may have some therapeutic effect *in vitro*, it is not clear how this relates to their efficacy *in vivo*. On the one hand, it is tempting to speculate that only the antibodies exhibiting near complete inhibition *in vitro* would be effective *in vivo* given the obstacle of the blood brain barrier and access to PrP in cells. However, it is also possible that only partial inhibition of conversion is required *in vivo* allowing the cells to “recover”. In either case, it would be advantageous for these therapeutic antibodies to have high affinities of binding to PrP^C^ and/or PrP^Sc^, as well as targeting specific critical PrP domains. One can hypothesize that the simultaneous targeting of more than one critical epitope will lead to greater benefits. However, co-treatment experiments performed with a mixture of two antibodies compatibly binding cell-surface PrP^C^ did not show any benefit with compared to treatment involving a single Mab in our current experiments. In a previous study [58], we demonstrated synergistic binding with one of our antibody pairs. Synergistic binding of inhibitory Mabs, i.e. reaction with an antibody that increases the binding of the second antibody, would be predicted to enhance the inhibitory effect. Further studies with antibody pairs fitting this description will be required to test this hypothesis. In addition, determining the significance of the Mab’s ability to bind both PrP^C^ and PrP^Sc^ may provide further insight into the conversion process.

## References

[1] Caughey BW, Dong A, Bhat KS, Ernst D, Hayes SF, et al (1991) Secondary structure analysis of the scrapie-associated protein PrP 27–30 in water by infrared spectroscopy. Biochemistry. 30: 7672–7680. 10.1021/bi00245a003 1678278

[2] Gasset M, Baldwin MA, Fletterick RJ, Prusiner SB (1993) Perturbation of the secondary structure of the scrapie prion protein under conditions that alter infectivity. Proc Natl Acad Sci U S A 90: 1–5. 8419912 10.1073/pnas.90.1.1 PMC45587

[3] Pan KM, Baldwin M, Nguyen J, Gasset M, Serban A, et al (1993) Conversion of α-helices into β-sheets features in the formation of the scrapie prion proteins. Proc Natl Acad Sci U S A 90: 10962–10966. 7902575 10.1073/pnas.90.23.10962 PMC47901

[4] Safar J, Roller PP, Gajdusek DC, Gibbs CJ Jr (1993) Conformational transitions, dissociation, and unfolding of scrapie amyloid (prion) protein. J Biol Chem 268: 20276–20284. 8104185

[5] Colby DW, Prusiner SB (2011) Prions. Cold Spring Harb.Perspect. Biol3, a006833). DOI: 10.1101/cshperspect.a006833. 10.1101/cshperspect.a006833 PMC3003464 21421910

[6] Prusiner SB (1982) Novel proteinaceous infectious particles cause scrapie. Science 216: 136–144. 6801762 10.1126/science.6801762

[7] Enari M, Flechsig E, Weissmann C (2001) Scrapie prion protein accumulation by scrapie-infected neuroblastoma cells abrogated by exposure to a prion protein antibody. Proc Natl Acad Sci USA 98: 9295–9299. 11470893 10.1073/pnas.151242598 PMC55414

[8] Peretz D, Williamson RA, Kaneko K, Vergara J, Leclerc E, et al (2001) Antibodies inhibit prion propagation and clear cell cultures of prion infectivity. Nature 412: 739–743. 11507642 10.1038/35089090

[9] White AR, Enever P, Tayebi M, Mushens R, Linehan J, et al (2003) Monoclonal antibodies inhibit prion replication and delay the development of prion disease. Nature 422: 80–83. 12621436 10.1038/nature01457

[10] Sigurdsson EM, Sy MS, Li R, Scholtzova H, Kascsak RJ, et al (2003) Anti-PrP antibodies for prophylaxis following prion exposure in mice. Neurosci Lett 336: 185–187. 12505623 10.1016/s0304-3940(02)01192-8

[11] Beringue V, Vilette D, Mallinson G, Archer F, Kaisar M, et al (2004) PrPSc binding antibodies are potent inhibitors of prion replication in cell lines. J Biol Chem 279: 39671–39676. 15133046 10.1074/jbc.M402270200

[12] Pankiewicz J, Prelli F, Sy MS, Kascsak RJ, Kascsak RB, et al (2006) Clearance and prevention of prion infection in cell culture by anti-PrP antibodies. Eur J Neurosci 24: 2635–2647. 10.1111/j.1460-9568.2006.04805.x PMC1779824 16817866

[13] Wisniewski T, Goñi F (2012) Could immunomodulation be used to prevent prion diseases? Expert Rev Anti Infect Ther10: 307–317. 10.1586/eri.11.177 PMC3321512 22397565

[14] Horiuchi M, Caughey B (1999) Specific binding of normal prion protein to the scrapie form via a localized domain initiates its conversion to the protease-resistant state. EMBO J 18: 3193–3203. 10369660 10.1093/emboj/18.12.3193 PMC1171400

[15] Saborio GP, Permanne B, Soto C (2001) Sensitive detection of pathological prion protein by cyclic amplification of protein misfolding. Nature 411: 810–813. 11459061 10.1038/35081095

[16] Castilla J, Saa P, Hetz C, Soto C (2005) In vitro generation of infectious scrapie prions. Cell 121: 195–206. 15851027 10.1016/j.cell.2005.02.011

[17] Castilla J, Saa P, Morales R, Abid K, Maundrell K, et al (2006) Protein misfolding cyclic amplification for diagnosis and prion propagation studies. Methods Enzymol 412: 3–21. 17046648 10.1016/S0076-6879(06)12001-7

[18] Shikiya RA, Ayers JI, Schutt CR, Kincaid AE, Bartz JC (2010) Coinfecting prion strains compete for a limiting cellular resource. J Virol 84: 5706–5714. 20237082 10.1128/JVI.00243-10 PMC2876617

[19] Kim NH, Choi JK, Jeong BH, Kim JI, Kwon MS, et al (2005) Effect of transition metals (Mn, Cu, Fe) and deoxycholic acid (DA) on the conversion of PrPC to PrPres. FASEB J 19: 783–785. 15758042 10.1096/fj.04-2117fje

[20] Nishina KA, Deleault NR, Mahal SP, Baskakov I, Luhrs T, et al (2006) The stoichiometry of host PrPC glycoforms modulates the efficiency of PrPSc formation in vitro. Biochemistry 45: 14129–14139. 17115708 10.1021/bi061526k

[21] Murayama Y, Yoshioka M, Yokohama T, Iwamaru Y, Imamura M, et al (2007) Efficient in vitro amplification of a mouse-adapted scrapie prion protein. Neurosci Lett 413: 270–273. 17174030 10.1016/j.neulet.2006.11.056

[22] Deleault NR, Harris BT, Rees JR, Supattapone S (2007) Formation of native prions from minimal components in vitro. Proc Natl Acad Sci USA 104: 9741–9746. 17535913 10.1073/pnas.0702662104 PMC1887554

[23] Mays CE, Titlow W, Seward T, Telling GC, Ryou C (2009) Enhancement of protein misfolding cyclic amplification by using concentrated cellular prion protein source. Biochem Biophys Res Commun 388: 306–310. 19664595 10.1016/j.bbrc.2009.07.163 PMC2756978

[24] Kim JI, Surewicz K, Gambetti P, Surewicz WK (2009) The role of glycophosphatidylinositol anchor in the amplification of the scrapie isoform of prion protein in vitro. FEBS Lett 583: 3671–3675. 19854187 10.1016/j.febslet.2009.10.049 PMC2856614

[25] Abid K, Morales R, Soto C (2010) Cellular factors implicated in prion replication. FEBS Lett 584: 2409–2414. 20412808 10.1016/j.febslet.2010.04.040 PMC2898186

[26] Mays CE, Ryou C (2010) Plasminogen stimulates propagation of protease resistant prion protein in vitro. FASEB J 24: 5102–5112. 20732953 10.1096/fj.10-163600

[27] Soto C, Anderes L, Suardi S, Cardone F, Castilla J, et al (2005) Presymptomatic detection of prions by cyclic amplification of protein misfolding. FEBS Lett 579: 638–642. 15670821 10.1016/j.febslet.2004.12.035

[28] Gonzalez-Romero D, Barria MA, Leon P, Morales R, Soto C (2008) Detection of infectious prions in urine. FEBS Lett 582: 3161–3166. 18706416 10.1016/j.febslet.2008.08.003 PMC2593137

[29] Jones M, Peden AH, Prowse CV, Groner A, Manson JC, et al (2007) In vitro amplification and detection of variant Creutzfeldt-Jakob disease PrPSc. J Pathol 213: 21–26. 17614097 10.1002/path.2204

[30] Atarashi R, Moore RA, Sim VL, Hughson AG, Dorward DW, et al (2007) Ultrasensitive detection of scrapie prion protein using seeded conversion of recombinant prion protein. Nat Methods 4: 645–650. 17643109 10.1038/nmeth1066

[31] Thorne L, Terry LA (2008) In vitro amplification of PrPSc derived from the brain and blood of sheep infected with scrapie. J Gen Virol 89: 3177–3184. 19008409 10.1099/vir.0.2008/004226-0

[32] Haley NJ, Mathiason CK, Zabel MD, Telling GC, Hoover EA (2009) Detection of sub-clinical CWD infection in conventional test-negative deer long after oral exposure to urine and feces from CWD+ deer. PLoS ONE 4: e7990. 19956732 10.1371/journal.pone.0007990 PMC2776529

[33] Rubenstein R, Chang B, Gray P, Piltch M, Bulgin MS, et al (2010) A novel method for preclinical detection of PrPSc in blood. J Gen Virol 91: 1883–1892. 20357038 10.1099/vir.0.020164-0

[34] Murayama Y, Yoshioka M, Masujin K, Okada H, Iwamaru Y, et al (2010) Sulfated dextrans enhance in vitro amplification of bovine spongiform encephalopathy PrPSc and enable ultrasensitive detection of bovine PrPSc. PLoS ONE 5: e13152. 20957174 10.1371/journal.pone.0013152 PMC2949392

[35] Hilmert H, Diringer H (1984) A rapid and efficient method to enrich SAF-protein from scrapie brains of hamsters. Bioscience Reports 4: 165–170. 6143576 10.1007/BF01120313

[36] Guo JP, Petric M, Campbell W, McGeer PL (2004) SARS corona virus peptides recognized by antibodies in the sera of convalescent cases. Virology 324: 251–256. 15207612 10.1016/j.virol.2004.04.017 PMC7125913

[37] Jiménez-Huete A, Lievens PMJ, Vidal R, Piccardo P, Ghetti B, et al (1998) Endogenous proteolytic cleavage of normal and disease-associated isoforms of the human prion protein in neural and non-neural tissues. Am J Pathol 153: 1561–1572. 9811348 10.1016/S0002-9440(10)65744-6 PMC1853409

[38] Rubenstein R, Kascsak RJ, Papini M, Kascsak R, Carp RI, et al (1999) Immune surveillance and antigen conformation determines humoral immune response to the prion protein immunogen. J Neurovirol 5: 401–413. 10463862 10.3109/13550289909029481

[39] Brown P (2002) Drug therapy in human and experimental transmissible spongiform encephalopathy. Neurology 58: 1720–1725. 12088013 10.1212/wnl.58.12.1720

[40] Supattapone S, Nishina K, Rees JR (2002) Pharmacological approaches to prion research. Biochem Pharmacol 63: 1383–1388. 11996878 10.1016/s0006-2952(02)00874-2

[41] Nunziante M, Gilch S, Schatzl HM (2003) Prion diseases: From molecular biology to intervention strategies. Chembiochem 4: 1268–1284. 14661267 10.1002/cbic.200300704

[42] Cashman NR, Caughey B (2004) Prion diseases–close to effective therapy? Nat Rev Drug Discov 3: 874–884. 15459678 10.1038/nrd1525

[43] Sim VL, Caughey B (2009) Recent Advances in Prion Chemotherapeutics. Infect Disord Drug Targets 9: 81–91. 19200018 10.2174/1871526510909010081 PMC3347484

[44] Priola SA, Raines A, Caughey WS (2000) Porphyrin and phthalocyanineantiscrapie compounds. Science 287: 1503–1506. 10688802 10.1126/science.287.5457.1503

[45] Rudyk H, Vasiljevic S, Hennion RM, Birkett CR, Hope J, et al (2000) Screening Congo Red and its analogues for their ability to prevent the formation of PrP-res in scrapie-infected cells. J Gen Virol 81: 1155–1164. 10725446 10.1099/0022-1317-81-4-1155

[46] Sigurdsson EM, Brown DR, Daniels M, Kascsak RJ, Kascsak R, et al (2002) Immunization delays the onset of prion disease in mice. Am J Pathol 161: 13–17. 12107084 10.1016/S0002-9440(10)64151-X PMC1850699

[47] Kim CL, Karino A, Ishiguro N, Shinagawa M, Sato M, et al (2004) Cell-surface retention of PrPC by anti-PrP antibody prevents protease-resistant PrP formation. J Gen Virol 85: 3473–3482. 15483265 10.1099/vir.0.80113-0

[48] Perrier V, Solassol J, Crozet C, Frobert Y, Mourton-Gilles C, et al (2004) Anti-PrP antibodies block PrPSc replication in prion-infected cell cultures by accelerating PrPC degradation. J Neurochem 89: 454–463. 15056288 10.1111/j.1471-4159.2004.02356.x PMC2063508

[49] Heppner FL, Musahl C, Arrighi I, Klein MA, Rulicke T, et al (2001) Prevention of scrapie pathogenesis by transgenic expression of anti-prion protein antibodies. Science 294: 178–182. 11546838 10.1126/science.1063093

[50] Scott M, Groth D, Foster D, Torchia M, Yang SL, et al (1993) Propagation of prions with artificial properties in transgenic mice expressing chimeric PrP genes. Cell 73: 979–988. 8098995 10.1016/0092-8674(93)90275-u

[51] Priola SA, Chesebro B (1995) A single hamster PrP amino acid blocks conversion to protease-resistant PrP in scrapie-infected mouse neuroblastoma cells. J Virol 69: 7754–7758. 7494285 10.1128/jvi.69.12.7754-7758.1995 PMC189717

[52] Kocisko DA, Priola SA, Raymond GJ, Chesebro B, Lansbury PT Jr, et al (1995) Species specificity in the cell-free conversion of prion protein to protease-resistant forms: A model for the scrapie species barrier. Proc Natl Acad Sci USA 92: 3923–3927. 7732006 10.1073/pnas.92.9.3923 PMC42074

[53] Priola SA, Chabry J, Chan K (2001) Efficient conversion of normal prion protein (PrP) by abnormal hamster PrP is determined by homology at amino acid residue 155. J Virol 75: 4673–4680. 11312338 10.1128/JVI.75.10.4673-4680.2001 PMC114221

[54] Rigter A, Langeveld JPM, Timmers-Parohi D, Jacobs JG, Moonen PLJM, et al (2007) Mapping of possible prion protein self-interaction domains using peptide arrays. BMC Biochemistry8: 6 doi: 10.1186/1471–2091–86. 10.1186/1471-2091-8-6 PMC1855927 17430579

[55] Moroncini G, Kanu N, Solforosi L, Abalos G, Telling GC, et al (2004) Motif-grafted antibodies containing the replicative interface of cellular PrP are specific for PrPSc. Proc Natl Acad Sci U S A 101: 10404–10409. 15240877 10.1073/pnas.0403522101 PMC478584

[56] Feraudet C, Morel N, Simon S, Volland H, Frobert Y, et al (2005) Screening of 145 anti-PrP monoclonal antibodies for their capacity to inhibit PrPSc replication in infected cells. J Biol Chem 280: 11247–11258. 15618225 10.1074/jbc.M407006200

[57] Sadowski MJ, Pankiewicz J, Prelli F, Scholtzova H, Spinner DS, et al (2009) Anti-PrP Mab 6D11 suppresses PrP^Sc^ replication in prion infected myeloid precursor line FDC-P1/22L and in the lymphoreticular system in vivo. Neurobiol Dis 34: 267–278. 19385058 10.1016/j.nbd.2009.01.013 PMC2713020

[58] Chang B, Miller MW, Bulgin MS, Sorenson-Melson S, Balachandran A, et al (2008) PrP antibody binding-induced epitope modulation evokes immunocooperativity. J Neuroimmunol 205: 94–100. 18977037 10.1016/j.jneuroim.2008.09.013 PMC2645591

